# BoHV‐1 affects abortion and progesterone in dairy cows Bovine alphaherpesvirus 1 (BoHV‐1) seropositivity, progesterone levels and embryo loss of 30‐day‐old pregnant dairy cows in Zagros Industrial Dairy Farm in Shahrekord: Examination and analysis

**DOI:** 10.1002/vms3.1187

**Published:** 2023-07-04

**Authors:** Amirhosein Khaneabad, Taghi Taktaz, Sajad Goodarzi, Hassan Momtaz

**Affiliations:** ^1^ Faculty of Veterinary Medicine, Department of Animal Clinical Science, Shahrekord Branch Islamic Azad University Shahrekord Iran; ^2^ Faculty of Veterinary Medicine, Department of Microbiology, Shahrekord Branch Islamic Azad University Shahrekord Iran

**Keywords:** BoHV‐1, cattle, embryo loss, progesterone, seroprevalence

## Abstract

**Background:**

Bovine alphaherpesvirus 1 (BoHV‐1) is a serious disease with severe negative economic effects on the global cattle sector, especially in Iran.

**Objective:**

This cross‐sectional study was carried out to examine the seroprevalence and associated risk factors of BoHV‐1 infection with progesterone levels and embryo death in 30‐day pregnant dairy cattle at Zagros Industrial Dairy Farm in Shahrekord, Iran.

**Methods:**

Between December 2017 to February 2018, blood samples were obtained from 60 dairy cow herds. To determine whether BoHV‐1 was present, serum samples were examined using the ELISA for serum antibodies. To find progesterone (P4) in blood, the progesterone ELISA test was used.

**Results:**

96.7 % of sera tested positive for BoHV‐1 antibodies, according to the findings. Additionally, 60.34 % of blood samples that tested positive had an experience of abortion and significantly more inseminations that resulted in pregnancy, consistent with findings from other studies conducted in Iran and other nations.

**Conclusions:**

Since this study is the first to document the risk factor for BoHV‐1 infection in Shahrekord, Iran, we could infer that the virus is extensively dispersed in this area.

## Graphical abstract

HIGHLIGHTS

1. This report presents the association between Progesterone, *BoHV‐1* and abortion in dairy cows.

2. Seropositivity of the *BoHV‐1* was related to abortion.

3. *BoHV‐1* is recognised as one of the most important causes of reproductive disorders.

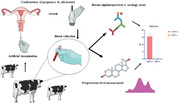

## 1 INTRODUCTION

Cattle herd efficiency has been proven to be critically dependent on herd health, especially viral infections (Hashemi et al., 2022). In the cattle industry worldwide, particularly in Iran, Bovine alphaherpesvirus 1 (BoHV‐1) has been regarded as the principal disease with considerable economic effects (Noaman et al., 2020). BoHV‐1 is the source of the highly contagious respiratory illness known as infectious bovine rhinotracheitis (IBR), accompanied by severe upper respiratory tract inflammation (Wathes et al., 2020). IBR is a widespread infectious disease that is quite common worldwide. The World Organization for Animal Health (OIE) must issue a statement when disease outbreaks occur in IBR‐free member states or IBR‐free zones of European Union countries due to the disease's significance for public health and the international trade of animals and animal products. Although some nations have eradicated IBR, the condition is still prevalent in dairy herds worldwide, including in Britain and Ireland (Iscaro et al., 2021). BoHV‐1 is a double‐stranded DNA virus with three subtypes and a member of the Herpesviridae family of viruses (Biswas et al., 2013). Recently, two BoHV‐1 subtypes were identified. Subtype 1 contains strains that cause respiratory conditions, including IBR, primarily determined by the development of exudative rhinotracheitis in the main bronchi of infected animals. The strains that cause infectious pustular vulvovaginitis and infectious balanoposthitis are part of subtype 2 (Newcomer et al., 2016).

Clinical BoHV‐1 infection symptoms, such as respiratory syndromes, genital infections, and neurological problems, depend on the characteristics of different subtypes (Graham., 2013). BoHV‐1 is spread by contact with mucosal droplets from infected animals (Marin et al., 2020). After the initial infection, the virus may stay dormant in some nervous system ganglia and escape after being reactivated. Along with impacting the health and welfare of animals, it also results in significant production losses because of higher indirect expenses related to veterinarian diagnosis, decreased dairy output, weight loss and mortality. Since BoHV‐1 may infect various hosts, it significantly impacts the animal trade and costs the international cattle business more than $3 billion yearly (Marin et al., 2020; Miller et al, 1985).

Pregnancy loss is a significant contributor to dairy cow infertility and may be the biggest source of financial loss in the current dairy industry (De Vries et al., 2006). Cattle after insemination (AI) have a high fertilisation success rate (90%), but their calving rates are much lower (30−50%), indicating that there were considerable embryonic losses throughout pregnancy (De Vries et al., 2006). When referring to the bovine species, the term ‘embryo’ refers to the development that occurs up to around 42−45 days following conception, during which time the cells are still differentiating (Jones et al., 2010). The term ‘embryonic mortality’ refers to the losses between fertilisation and the end of the differentiation stage, which is about day 40 (Alfieri et al., 2019). Therefore, this time frame is utilised to assess the rate of embryonic death in beef and dairy cattle (Parmar et al., 2016). Cattle embryonic mortality is a complex phenomenon that may be influenced by both genetic and environmental variables (Jones et al., 2010). However, infectious agents, including viruses, bacteria, and parasites, are the main reason dairy cattle are infertile. These substances can cause anestrus, repeated pregnancy, early embryonic death, retained placenta, delayed return to estrus, premature embryonic death and abortion (Abdalla et al., 2017). Except for fertilisation failure, roughly 40% of bovine embryos perish in the first 3 weeks following treatment or insemination, and cows resume estrus after 21−24 days (Carpenter et al., 2006; Noaman et al., 2020). Fertilisation failure and embryonic mortality, the latter of which is more important, are relatively well‐known phenomena that impact reproductive efficiency (Wathes et al., 2020). Early embryonic mortality is a substantial source of economic loss due to recurrent breeding and rising costs of artificial insemination (Alfieri et al., 2019).

Progesterone (P4) concentrations influence embryo survival before and after insemination (Nyman et al., 2018). There is convincing evidence that P4 concentrations at particular times directly correlate with pregnancy outcomes, both when they are excess and insufficient (Nyman et al., 2018). Luteal P4 is crucial for preserving an ideal uterine condition, preparing the uterus and oocyte before mating, and assisting the uterus in growing the embryo throughout gestation in the bovine (Fair et al., 2012). Variations in P4 during the luteal phase just before oestrus and after insemination may result in losses of embryos between days 4 through 8 following oestrus, during maternal detection of pregnancy on days 14 through 17, and throughout the late foetal stage from day 28 to days 42−50 (Inskeep et al., 2005). The frequency and pattern of pregnancy losses in dairy cattle have been extensively documented in publications. According to those mentioned above, the current study intended to investigate the impact of BoHV‐1 seropositivity on progesterone levels and embryo loss in dairy cows that were 30 days pregnant and kept at the Zagros Industrial Dairy Farm in Shahrekord.

## 2 MATERIALS AND METHODS

### 2.1 Study area and animals

From December 2017 to February 2018, this study was carried out at the Zagros Industrial Cattle Farm in Shahrekord. The region chosen represents well‐known milk sheds that supply milk to the country's major urban and peri‐urban areas. In this investigation, 60 cows whose pregnancies were determined by ultrasound 30 days after artificial insemination served as our target group. The cows were entirely Holstein breed and kept in intensive systems.

### 2.2 Blood collection and serological assay

The coccygeal vein was used as the site for blood collection, and disposable needles and simple vacutainer tubes were used. 10 mL of blood was taken from each animal randomly, stored overnight in an upright position, and then centrifuged at 1000 × *g* for 10 min. The serum samples were divided into clean cryovials, labelled and transferred on ice to the laboratory, where they were kept at −20°C until evaluation. According to the manufacturer's instructions, a Blocking immunoenzymatic assay (B‐ELISA) with 93% sensitivity and 99% specificity was used to identify antibodies specific for BoHV‐1 (INgezim IBR Compac, Ingenasa, Spain). In a BoHV‐1 antigen precoated plate, 50 μL of diluent (5‐chloro‐2‐methyl‐4‐isothiazolin‐3‐one and 2‐methyl‐4‐isothiazolin‐3‐one) and 50 μL of sera (test samples, negative and positive control) were added following the plate arrangement. The plate was incubated for an hour at 37°C and rinsed five times. 100 μL of the conjugate was added to each well, which was then incubated at 37°C for 30 min before being rinsed off. Finally, 100 μL of substrate solution was added and allowed to sit in a dark room for 15 min. To each well, 100 μL of stop solution was added. At 450 nm, the wells were investigated.

### 2.3 Progesterone assay

A commercial competitive ELISA was used to measure progesterone levels (ng/mL) (Monobind Inc., USA). The kit included 96‐well ELISA plates and the necessary reagents for analysing blood samples. The range of the standard curve was 0–60 ng/mL. The absorbance at 450 nm was measured using a BioTek ELx800 absorbance microplate reader (USA). The assay's minimal detection limit was 1 ng/mL, and the intra‐ and interassay relative standard deviations were 5.10% and 8.15%, respectively.

### 2.4 Statistical analysis

SPSS version 24 was used to statistical analysis the data. The relationship between progesterone level and BoHV‐1 seropositivity was examined using a *U* Mann–Whitney test. Variables that were considered to be significant and included in the final mode had a *p* value under 0.05.

## 3 RESULTS

In Shahrekord's dairy herds, the Zagros Industrial Cattle Farm's seroprevalence data against BoHV‐1 revealed a high overall seroprevalence of 58 positive (96.7%) and 2 negatives (3.3%) animals, according to Table 1. Also, the percentage of abortion in positive serum samples was reported as 15.51% (9 out of 58) and 0% (0 out of 2). Additionally, the results showed that the percentage and frequency distribution of cattle with a history of pregnancy loss was 35 (60.34 %) in seropositive samples and 0 (0 %) in seronegative samples.

**TABLE 1:** Frequency of general BoHV‐1 antibodies in the herds under study.

Results	*n*	(%)	Embryo loss (%)	History of abortion (%)
Positive	58	96.7	15.51	60.34
Negative	2	3.3	0	0
Total	60	100	100	100

Our results confirmed that more abortions occurred in IBR‐positive cows. Additionally, in these cows, embryo loss 30 days after artificial insemination was linked to a history of abortion. On day 30, there were no appreciable variations in the plasma P4 levels between seropositive and seronegative groups (Table 1). The results indicate no difference in the mean progesterone levels between the seropositive and seronegative sample groups (Table 2). By calculating the Spearman's correlation coefficient of the relationship between progesterone and IBR in cows that had positive IBR, the value of the correlation coefficient was 0.115. However, since the significance level of *p* value = 0.390 was obtained, it can be concluded that there is a significant relationship between progesterone and IBR (Table 3). Also, regardless of whether IBR was positive or negative, the correlation coefficient between all the cows was calculated, and the correlation coefficient was calculated as 0.104. According to the *p* value = 0.430, it can be concluded that there is no significant relationship between progesterone and IBR (Table 3)

**TABLE 2:** Correlation test results between progesterone and IBR on the 30th day of pregnancy.

	Correlation coefficient	Sig. (2‐tailed)	*N*
IBR positive	0.115	0.390	58
IBR negative	‐	‐	‐
Total	0.104	0.430	60

**TABLE 3:** Descriptive results of the amount of progesterone on the 30th day of pregnancy based on the abortion of the tested cows.

	*N*	Minimum	Maximum	Mean	SD	Mean ± SD
Nonaborted	51	1.17	7.09	2.972	1.720	2.972 ± 1.720
Aborted	9	3.88	9.42	5.740	2.390	5.740 ± 2.390
Total	60	1.17	9.42	3.387	2.068	3.387 ± 2.068

Mann–Whitney test statistic was used to compare the amount of progesterone. The value of *U* = 79 was obtained since the significance level of *p* value = 0.002 was accepted, so there is a significant difference between the amount of progesterone in the blood of cows that had an abortion and cows that did not have an abortion (Table 4).

**TABLE 4:** The results of the *U* Mann–Whitney test compare the mean ranks of the progesterone amount on the 30th day of pregnancy.

		*N*	Mean rank	Sum of ranks	Mann–Whitney *U*	Sig.
Progesterone	Aborted	51	27.55	1405.00	79.00	0.002
	Nonaborted	9	42.22	425.00		

## 4 DISCUSSION

BoHV‐1 is a highly contagious virus disseminated widely in bovine herds worldwide (Ata et al., 2012). BoHV‐1 threatens the cattle industry in several ways, mainly in terms of fertility (Lucchese et al., 2016). As a result, the livestock and dairy cow businesses suffer significant output losses (Ata et al., 2012). Up to this point, several studies have been conducted to determine the connection between BoHV‐1 infections and issues with cow infertility. A total of 624 blood samples from cows with reproductive issues were gathered by Abalar and Akça, who used a micro‐neutralisation technique to investigate the presence of BHV antibodies (Çabalar et al., 1994). They revealed that 425 (68.1%) tested positive for BoHV‐1 antibodies, suggesting that the virus may cause cows' reproductive issues because it is highly prevalent in herds. In this investigation, the prevalence of BoHV‐1 in cattle was 96.7% (Çabalar et al., 1994). Seroprevalence rates in cattle and farms worldwide are 12–77.5% and 43–100%, respectively (Raaperi et al., 2014). High seroprevalence at the farm level indicates that every dairy herd had at least one positive animal. The Zagros Industrial Cattle Farm in Shahrekord, Iran, had a high incidence of BoHV‐1 infection. In Iran, the total BoHV‐1 seroprevalence was reported at 31.9%, which is lower than the results of the current study (Nikbakht et al., 2015). Similar findings have been written by Kermam and Isfahan (Sakhaei et al., 2009; Shirvani et al., 2012). As a results establishing preventative and control measures between animals from this region and other regions is required because healthy cattle can get infected either directly via secretions (respiratory, ocular or reproductive) or indirectly through equipment or people (Ackermann., 2006).

Another goal of this study was to investigate the relationship between progesterone levels and abortion in BoHV‐1‐infected cows. In this study, reproductive losses after artificial insemination in cattle were evaluated. The results showed that the calving rate in cows whose pregnancy was confirmed by ultrasound was reported to be 86.2%. Foetal loss, which accounted for the most significant number of pregnancy losses in the present study, was estimated at 13.8%. All of them were confirmed in terms of the positivity of blood serum. In addition, the distribution of virus‐infected cows with a history of abortion was 60.34%. The results support earlier studies showing a high correlation between abortion and the seroprevalence of BoHV‐1. They discovered that cows with a history of abortion were more likely to get the virus than nonaborted animals (Wedajo et al., 2021).

A positive ELISA test indicates that an animal was exposed to the BoHV‐1 virus throughout its lifespan. However, as it is not a direct diagnostic method, it cannot establish that the virus was the main cause of the miscarriage (Selim et al., 2022). The main hypothesis of this study was that the BoHV‐1 virus could lead to foetal loss through the effect on P4 levels. A favourable outcome after artificial insemination appears to be significantly influenced by P4 levels throughout the oestrous cycle (Diskin et al., 2016). It was amply demonstrated that the progesterone concentration on the day of prostaglandin‐induced luteolysis and the subsequent embryo survival rate had a positive linear relationship (Diskin et al., 2016). There were no noticeable variations in the levels of P4 between the seropositive and seronegative groups during gestation in the current study. But compared to cattle that did not have abortions, the mean P4 in cattle who underwent abortions following study‐related sample collection revealed a significant difference. This research showed that the amount of P4 in cows that did not have an abortion was lower than that of cows that experienced an abortion.

Foetal death has been demonstrated as a risk factor connected to the manifestation of IBR. This could be the case because, following viraemia, the virus passes through the placenta and into the foetus's bloodstream, lethally infecting it, killing it and then expelling it (Ortiz‐González et al., 2022). Although the path used by the virus to reach the foetus from the placenta is uncertain, persistent viral lesions found in the foetal liver point to the umbilical vein as the site of haematogenous spread (Ortiz‐González et al., 2022). Based on the findings of this research, a high percentage of the studied samples were IBR positive, which can be related to abortion in IBR‐positive cows.

In line with the study, Sibhat et al. (2018) showed that BoHV‐1 seroprevalence is very high in herds and animals in Ethiopia. The discovery of seropositive cows in every location under investigation demonstrated the country's viral expansion. According to prior research, the critical risk factors for the presence of BoHV‐1 among cow farms are big herd sizes, bought livestock, older age, high herd densities within a region, dairy cattle farms with the presence of beef cattle, and dairy cattle farms next to BoHV‐1 positive farms (Almeida et al., 2013). Similar to this, other management practices, such as the presence of cattle from various herds, the purchase of animals, and the use of uncertified semen, impact how the illness manifests and spreads within the herd. As a result, it is crucial to execute and create new IBR prevention and control strategies at the national level (Almeida et al., 2013).

## 5 CONCLUSION

In summary, the findings of this study show the potential significance of BoHV‐1 as a causative factor of reproductive disorders in cattle in Sharekord Province, one of Iran's most significant dairy cow production regions. These viral disorders' clinical relevance must be thoroughly studied through isolations from aborted foetuses or evidence of current infection. Due to the rise in the cost of production resulting from veterinary diagnosis, treatment of clinical symptoms, and the loss of virus‐carrying animals, the high incidence of IBR in this area might be detrimental to many farms. Additionally, a strong correlation between the disease and the existence of reproductive abnormalities was discovered, proving that this virus may damage cattle and result in abortions and foetal deaths in these animals. According to this study, a comprehensive surveillance and control approach is also required to lower the prevalence of BoHV‐1 infection at Zagros Industrial Cattle Farm.

## AUTHOR CONTRIBUTIONS

AK and SG were responsible for sample collection. TT and HM participated in the design of the study and performed the statistical analysis and writing the manuscript. TT and AK are responsible for doing the whole project and submitting the article. All authors have read and approved the final version of this manuscript for publication.

## FUNDING

The authors received no financial support for the research, authorship and/or publication of this article. No particular grant was given to this research by funding organisations in the public, private or not‐for‐profit sectors.

## CONFLICT OF INTEREST STATEMENT

The authors declare no conflicts of interest.

### ETHICS STATEMENT

The authors certify that the ethical policies of the journal, as outlined on the journal's author guidelines page, have been followed and the approval of the appropriate ethical review committee has been obtained. This investigation was conducted in accordance with Iran's guidelines for the care and use of animals.

### PEER REVIEW

The peer review history for this article is available at https://publons.com/publon/10.1002/vms3.1187.

## Data Availability

All data analyzed during this study are included in this published article.
